# Meeting report of the fifth symposium organized by the coalition to strengthen the HPV immunization community (CHIC): Nairobi, Kenya – advancing sustainable HPV vaccination programs in Africa

**DOI:** 10.1186/s12919-026-00403-2

**Published:** 2026-10-26

**Authors:** Ana Bolio, Mary Carol Jennings, Ricardo Burdier, Sakshi Jindal, Shana Kagan, Emilie Karafillakis, Elaine Chaurat, Andreas Ateke Njoh, Edina Amponash-Dacosta, Sambo Guemgo, Renée Didace Bakouan, Mbye Njie, Rose E. Jalang’o, Christine Wangeci, Rosemary Njura Njogu, Alassane Ndiaye, Clara Mashiringo, Christopher Morgan, Alex Vorsters, Dur-e-Nayab Waheed

**Affiliations:** 1 The Vaccine Confidence Project, London School of Hygiene & Tropical Medicine, London, UK; 2 Jhpiego, Baltimore, MD USA; 3 Centre for Evaluation of Vaccination, Vaccine and Infectious Disease Institute, University of Antwerp, Antwerp, Belgium; 4 Expanded Program On Immunization, Ministry of Health, Yaoundé, Cameroon; 5 School of Global Health and Bioethics, Euclid University, Bangui, Central African Republic; 6 Vaccines for Africa Initiative and NITAG Support Hub, School of Public Health, Faculty of Health Sciences, University of Cape Town, Cape Town, South Africa; 7 World Health Organization - AFRO/UCN/UHC/DPC/Vaccine-Preventable Diseases Inter-Country Support Team for West and Central Africa, Brazaville, Congo; 8 Expanded Program On Immunization, Ministry of Health, Ouagadougou, Burkina Faso; 9 Expanded Program On Immunization, Ministry of Health, Banjul, Gambia; 10 National Vaccines & Immunization Program, Ministry of Health, Nairobi, Kenya; 11 Jhpiego, Nairobi, Kenya; 12 Immunisation Division, Ministry of Health and Public Hygiene, Dakar, Senegal; 13 Ministry of Health and Child Care, Harare, Zimbabwe

**Keywords:** HPV vaccine, Cervical cancer prevention, Africa, HPV vaccine implementation

## Abstract

HPV vaccination coverage across Africa has improved substantially in recent years but remains below elimination thresholds, with persistent gaps in reaching hard-to-reach populations and growing uncertainty around future financing. This report summarizes proceedings from the fifth regional symposium of the Coalition to Strengthen the HPV Immunization Community (CHIC), held in Nairobi, Kenya, in October 2025 in collaboration with Kenya's Ministry of Health. The three-day symposium convened over 100 participants from 22 African countries, including national immunisation program managers, ministry officials, researchers, civil society representatives, and global partners (WHO, UNICEF, Gavi, PATH, the Gates Foundation). The agenda, developed through stakeholder interviews and prior scientific and advisory input, centered on six subthemes related to program sustainability. Plenary sessions addressed epidemiological trends, national policy updates including Kenya's transition to a single-dose schedule, and implications of Gavi's upcoming consolidated funding model (Gavi 6.0). Country snapshot presentations and service-delivery case studies facilitated cross-country peer learning. On day three, though an electronic poll of helped to prioritize 12 candidate implementation themes, which structured subsequent round table working groups. Countries then developed individual action plans identifying priority challenges, responsible stakeholders, and implementation timelines. Sustainable service delivery, financing, and data systems emerged as the highest-priority implementation themes. Country action plans most frequently targeted data systems strengthening, communication and community engagement, sustainable service delivery, and financing mechanisms, with additional attention to service integration, hard-to-reach populations, and target population expansion. Cross-cutting synthesis identified recurring concerns regarding HPV vaccination for people living with HIV, fragmented data systems, the role of program integration, financing sustainability under evolving donor models, and underrecognized risks from misinformation, including emerging AI-generated content. The symposium reinforced CHIC's role as a platform for peer-to-peer learning across countries at varying stages of HPV vaccine introduction, surfacing shared structural barriers to sustainability alongside country-specific action plans. Findings underscore the need for sustained political commitment, financing transition planning, digital system strengthening, and multisectoral integration to support the WHO goal of cervical cancer elimination by 2030.

**Supplementary Information:**

The online version contains supplementary material available at https://doi.org/10.1186/s12919-026-00403-2.

## Introduction

**Fig. 1:**
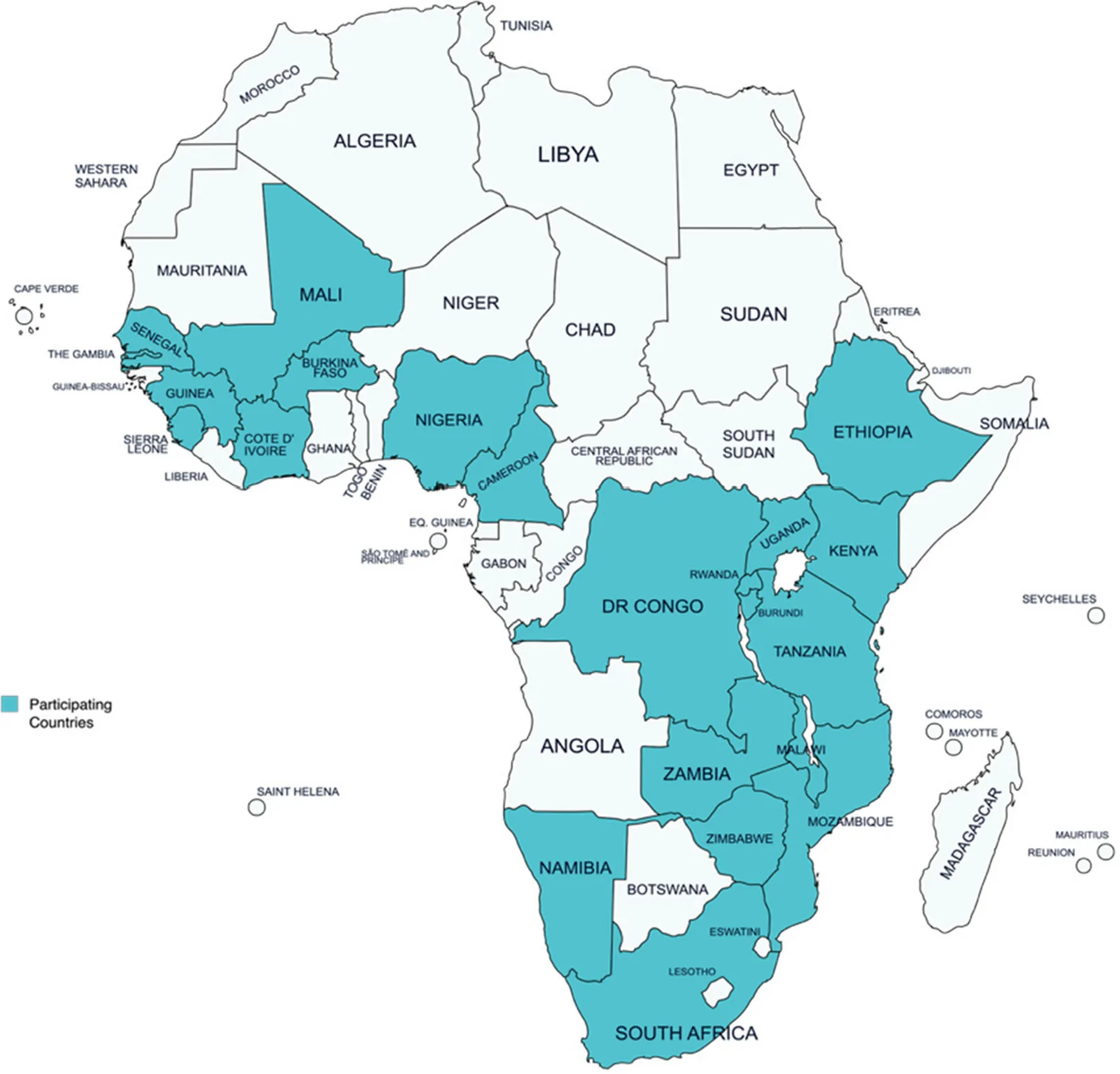
Countries represented during the Nairobi symposium participants

We report on the proceedings of a three-day Symposium on *Advancing Sustainable HPV Vaccination Programs in Africa*, held in Nairobi, Kenya in October 2025 in collaboration with the Kenya Ministry of Health, convening more than 100 participants from 22 African countries (Fig. 1). Participants included national immunization program managers and other EPI (Essential Program on Immunization) staff, leadership from ministries of health and education, policymakers, technical experts, researchers, and representatives from civil society, academic institutions, technical working groups, and global partners such as the World Health Organization (WHO), the United Nations Children’s Fund (UNICEF), Gavi, the Vaccine Alliance, the Program for Appropriate Technology in Health (PATH), and the Gates Foundation.

The symposium was organised by the Coalition to Strengthen the HPV Immunization Community (CHIC) [1]: a partnership led by a Secretariat convened between the Centre for the Evaluation of Vaccination at the University of Antwerp, Jhpiego, and the Vaccine Confidence Project at the London School of Hygiene & Tropical Medicine. This event built on four previous regional symposia held in Addis Ababa (2022), Delhi (2022) [2], Abidjan (2023) [3], and Manila (2024), reflecting a growing regional community of practice and an evolving evidence base in HPV vaccine implementation across low- and middle-income country settings. Kenya’s Ministry of Health played a central role in planning and hosting this fifth symposium, marking the country’s leadership in advancing global dialogue on HPV vaccine uptake and equity.

Regional evidence indicates that, at the time of this Symposium, 95% of Eastern and Southern African (ESA) countries (19/20) had introduced HPV vaccination, with 74% adopting the single-dose schedule. Coverage in ESA increased from 52% in 2021 to 74% in 2024, reflecting post-COVID recovery, yet remaining below the 90% elimination threshold. In West and Central Africa, 16 of 27 countries had introduced HPV vaccination, with Burkina Faso and Cabo Verde exceeding 99% coverage. Large-scale multi-age cohort (MAC) campaigns demonstrated substantial reach, including 12.3 million girls vaccinated in Nigeria (~ 86%) and over 90% coverage in Malawi and Mozambique multi-age campaigns. These achievements reinforce both the feasibility and vulnerability of sustaining progress.

### Agenda and participants

The agenda for this fifth symposium was developed through a structured participatory engagement process that characterizes CHIC’s approach to country-led engagement across our community of practice [4, 5]. Our Secretariat conducted twenty-five stakeholder consultations in addition to scientific and advisory group meetings, which highlighted the community of practice’s priority to engage on how to sustain human papillomavirus (HPV) programs. The process by which this stakeholder input was gathered and synthesized into the symposium agenda is summarized in Additional file 1.

We synthesised this formative stakeholder input into six subthemes that shaped the agenda, all relating to the overarching theme of fostering program sustainability:

1. Searching for the most efficient local mix of school, facility and community-based point of care delivery;
2. Looking for strategies to raise new domestic funds to sustain HPV vaccination programs;
3. Looking for good practices to combine trusted methods with new technologies to overcome hesitancy and build trust;
4. Tailoring what works to reach adolescents who are out of school or otherwise hard to reach;
5. Being thoughtful about taking an integrated approach to HPV vaccination and other services and programs; and
6. Looking for insights to guide decision-making on whether or not to expand the focus group for HPV vaccines beyond the primary eligible population of girls aged 9 to 14 years.

In addition to featuring national experts, the symposium included a call for abstracts, which received over 50 submissions and supported 19 accepted presenters, marking the largest group of early- and mid-career researchers making such contributions to a CHIC event to date (Additional file 2 Table S1).

In order to foster peer-learning conversations from across a broad range of experience, this symposium brought together countries at varying stages of HPV vaccination program maturity, ranging from early adopters with years of experience to those still preparing for introduction. Our formative work identified a gap in peer-learning exchanges between francophone and anglophone countries, resulting in our intentional inclusion of representatives from both categories. We offered simultaneous translation and bilingual meeting materials to ensure inclusivity. Recognizing Africa's rich multilingual landscape, future symposia will explore opportunities to include additional languages, including Portuguese and other regionally appropriate languages, ensuring inclusive peer-learning exchanges.

### Overview of themes

The symposium fostered growth in communal understanding of current challenges and emerging developments in sustaining HPV vaccination programs, drawing on both scientific and programmatic perspectives. Over the course of the three-day meeting, participants engaged in cross-country dialogues to exchange practical experience on sustaining and strengthening routine delivery, and worked collaboratively to translate these insights into action-oriented roadmaps for sustainable HPV vaccination in their own country contexts.

Discussions highlighted work on addressing coverage gaps for out-of-school and hard-to-reach girls, the transition to single-dose vaccination schedules, and the delivery of vaccines in fragile or conflict-affected settings. Participants also focused on strengthening data systems and countering misinformation, and reflected on ways to incorporate HPV vaccination into broader adolescent and primary health care programming. Global partner contributions in particular informed cross-cutting discussions involving planning for increasing domestic investments in vaccination, as well as how countries might respond proactively to anticipated shifts in Gavi 6.0 financing [6], including what the transition to a new consolidated funding model means for the long-term sustainability of HPV programs, explored in greater detail below.

These discussions helped country representatives define medium-term priorities and identify opportunities for collaboration and targeted support, as described in detail below.

### Plenary sessions

The symposium opened with a welcoming statement from Kenya’s Ministry of Health, which underscored the urgency of accelerating HPV vaccination as part of the country’s broader commitment to cervical cancer elimination. The Ministry announced Kenya’s upcoming transition to a single-dose HPV schedule, beginning in October 2025, an evidence-driven decision that national leaders framed as essential for expanding access, reducing logistical barriers, and improving sustainability in the setting of constrained fiscal resources. In his remarks, the Director-General of Health emphasised that “no girl should be left behind,” calling for stronger collaboration with the Ministry of Education, community structures, and county governments to better reach girls in and out of school. This framing set the tone for the symposium: a collective commitment to equity, adaptation, and long-term sustainability across African programs.

#### Regional epidemiological and programmatic updates by the World Health Organization (WHO)

WHO global and regional teams highlighted the disproportionate burden of cervical cancer in the African region and the persistent gap between vaccine availability and coverage. Presenters highlighted how several high-incidence countries continue to have some of the lowest HPV vaccination rates, driven in part by challenges in reaching out-of-school girls, sustaining routine delivery, and countering misinformation. WHO speakers urged participating countries to maintain early prevention as a priority, strengthen routine immunization systems, and continuously invest in community-based demand generation, echoing concerns many participants shared throughout the meeting.

#### Gavi 6.0: implications for HPV vaccination

The agenda also allowed for a unique opportunity for Gavi, the Vaccine Alliance to engage the community of practice in discussions on the upcoming shift toward a consolidated funding model under the agency’s next five-year planning cycle (Gavi 6.0, 2026–30), which will merge multiple grant windows into a single application with expanded co-financing obligations. While participants welcomed the simplified structure, many expressed concerns about HPV competing for visibility within broader immunization budgets, particularly as overall resources are expected to decline given radical shifts in global health financing. Gavi speakers reaffirmed HPV vaccination as a core priority and encouraged governments to embed the HPV vaccine proactively into EPI microplanning, including school outreach, community mobilization, data systems, and cold chain requirements, to support long-term program sustainability within the new financing environment. Participants from Kenya shared the example that re-entering Gavi’s preparatory phase has allowed them more time to realign domestic resources for co-financing and local vaccine production efforts (e.g., Kenya BioVax [7]). Middle-income countries and transition-phase countries continue to face heightened sustainability pressures, requiring early integration of HPV vaccination into domestic health budgets and national immunization investment cases.

#### Country snapshots

The *Country snapshot* session is a core feature of CHIC symposia, offering a dedicated space for country participants to share where their countries stand on HPV vaccination programs, what is working, and what remains challenging. Country contingents had prepared concise posters (“snapshots”) profiling their HPV vaccination programs, recent coverage trends, delivery strategies, and current challenges (Table 1). Of the 19 countries with established programs, 16 reported a "mixed" delivery strategy, combining school-based, facility-based, and community-based platforms, while Rwanda, South Africa, and Uganda relied primarily on school-based delivery alone. Participants presented these in an interactive session to begin cross-country dialogues reflecting on current situations, and to begin surfacing common patterns across countries, such as the prevalent use of school-based delivery, the importance of coordinated microplanning with education authorities, and the need to map and enumerate special groups such as out-of-school girls. For countries yet to introduce the vaccine, snapshots focused on strategy roadmaps, funding expectations, and initial partner coordination needs. Together, these conversations reflected the kind of peer-to-peer exchange grounded in real programmatic experience that the CHIC platform is uniquely positioned to facilitate.

**Table 1:** Overview of HPV vaccination programs in participating countries (information current as of Dec 2025)

Country Name	HPV vaccine introduction year	Schedule (Year of transition to single dose)	Target cohort	Catch-up policy	Delivery strategy	Coverage (2024)	Future Plans
Burkina Faso	2022	One (2023)	Girls 9 years	NA	Mixed	99%	Expand the vaccine target cohort to 18 years
Burundi	Planned for 2026	One*	Girls 9–14*		Mixed*	NA	
Cameroon	2020	One (2023)	Girls and boys 9 years	Girls and boys 10–14 years	Mixed	31%	Regular vaccine supplies, scale up to other priority groups
Côte d'Ivoire	2019	One (2023)	Girls 9 years	Girls 10–14 years	Mixed	61%	Expand vaccine target cohort to girls 9–18 years. Improve target population estimates and data monitoring quality
Democratic Republic of the Congo	Planned for 2026		Girls 9 years*	Girls 9–14 years*	Mixed*	NA	
The Gambia	2019	One (2024)	Girls grade 3 and OOS 9–14 years	NA	Mixed	12%	Improve target population estimates based on mini census; Conduct rounds of annual school vaccination
Guinée	Planned for 2026	One*	Girls 9 years*	Girls 9–14 years*	Mixed*	NA	
Kenya	2019	One (2025)	Girls 10–14 years	NA	Mixed	79%	Increase coverage. Intensify demand generation Improve integration
Malawi	2019	One (2025)	Girls 9 years	Girls 9–14 years	Mixed	38%	Strengthen school-based delivery. Improve 2nd and 3rd dose uptake among HIV +
Mali	2024	One (Introduced as single dose)	Girls 10 years	NA	Mixed	15%	Expand vaccine target cohort to 9–14 years. Improve coverage
Namibia	2025	One (Introduced as single dose)	Girls 9–14 years	NA	Mixed	NA	Increase coverage, expand vaccine target population to older adolescents and boys
Nigeria	2023	One (Introduced as single dose	Girls 9 years	Girls 9–14 years	Mixed	60%	
Rwanda	2011	One (2024)	Girls 12 years	NA	School-based	86%	Strengthen collaboration with other institutes and stakeholder engagement
Senegal	2018	Two	Girls 9 years	Girls 10–14 years	Mixed	HPV1 66% HPVc 51%	Implementation of recovery plan to increase coverage
South Africa	2014	One (2024)	Girls in grades 5–7	NA	School-based	88%	
Uganda	2015	One (2025)	Girls 10 years	NA	School-based	95%	Ensure optimal delivery among immunocompromised Strengthen stakeholder engagement
Tanzania	2018	One (2025)	Girls 9 years	NA	Mixed	94%	
Zambia	2019	One (2023)	Girls 9 years	Girls 10–14 years	Mixed	60%	Improve coverage
Zimbabwe	2018	One (2025)	Girls in grade 5 and OOS 10 years	NA	Mixed	85%	Increase coverage

*OOS – out of school*

^*^ Indicates planned introduction

Source**:** Data compiled from country snapshots presented during the CHIC Symposium, available online [8], and from the World Health Organization (WHO) HPV Vaccine Dashboard, available online [9]

#### Service delivery adaptation and resilience

Country presentations on this topic illustrated adaptations of delivery models in response to evolving evidence, population dynamics, and operational realities. Notable examples include descriptions from Uganda and Rwanda of how strong partnerships between health and education sectors have facilitated effective school-based strategies, with Rwanda showcasing its national digital registry that allows real-time monitoring, improved accuracy, and automatic short message service (SMS) reminders for caregivers. Zimbabwean participants shared how peer mobilisation, district-level social mobilisation, and coordination with local leaders helped counter rumours and improve uptake in both urban and rural settings.

Kenyan participants highlighted the value of multisectoral collaboration through national and county technical working groups, involving health promotion teams, community health workers, faith-based organizations, and professional associations. Participants from Nigeria further emphasised how engaging traditional and religious leaders, especially in communities with low vaccine confidence, was instrumental in reaching millions of girls during its phased introduction.

Across countries, speakers echoed similar challenges: difficulties reaching out-of-school youth, nomadic populations, and those affected by conflict; health worker turnover; and misinformation, including fertility-related rumours. Several countries described perceived efficiency gains after switching to a single-dose schedule, including reduced logistical burdenand simpler cross-sectoral coordination with schools.

Together, these presentations reinforced a central emergent theme of the symposium: while delivery models differ by context, sustained coverage depends on strong partnerships, flexible strategies, and systems that adapt to shifting resource and population realities.

### Implementation working groups

#### Structure

The initial landscaping and peer exchange sessions were followed by a series of iterative working groups focused on challenges to HPV program implementation, to support participants in translating symposium learnings and peer experiences into actionable program insights for the future. Working groups were structured to allow participants to develop action-oriented “roadmaps for sustainable HPV vaccination” tailored to the local context of their country of origin.

### Theme selection

**Fig. 2:**
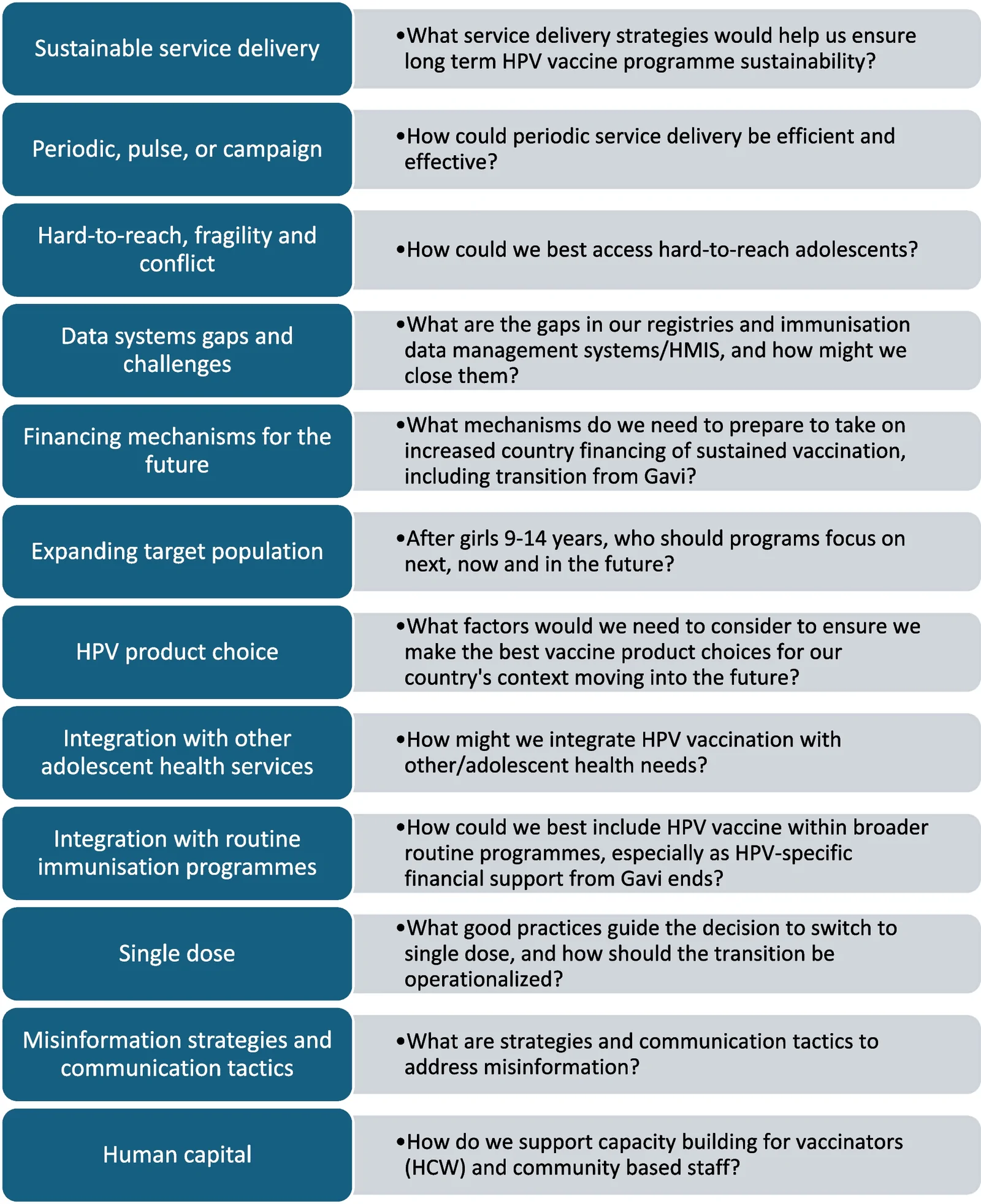
List of themes identified during the preparation phase and the first two days that were included in the poll. *HMIS – Health Management Information System, HCW – healthcare worker*

**Fig. 3:**
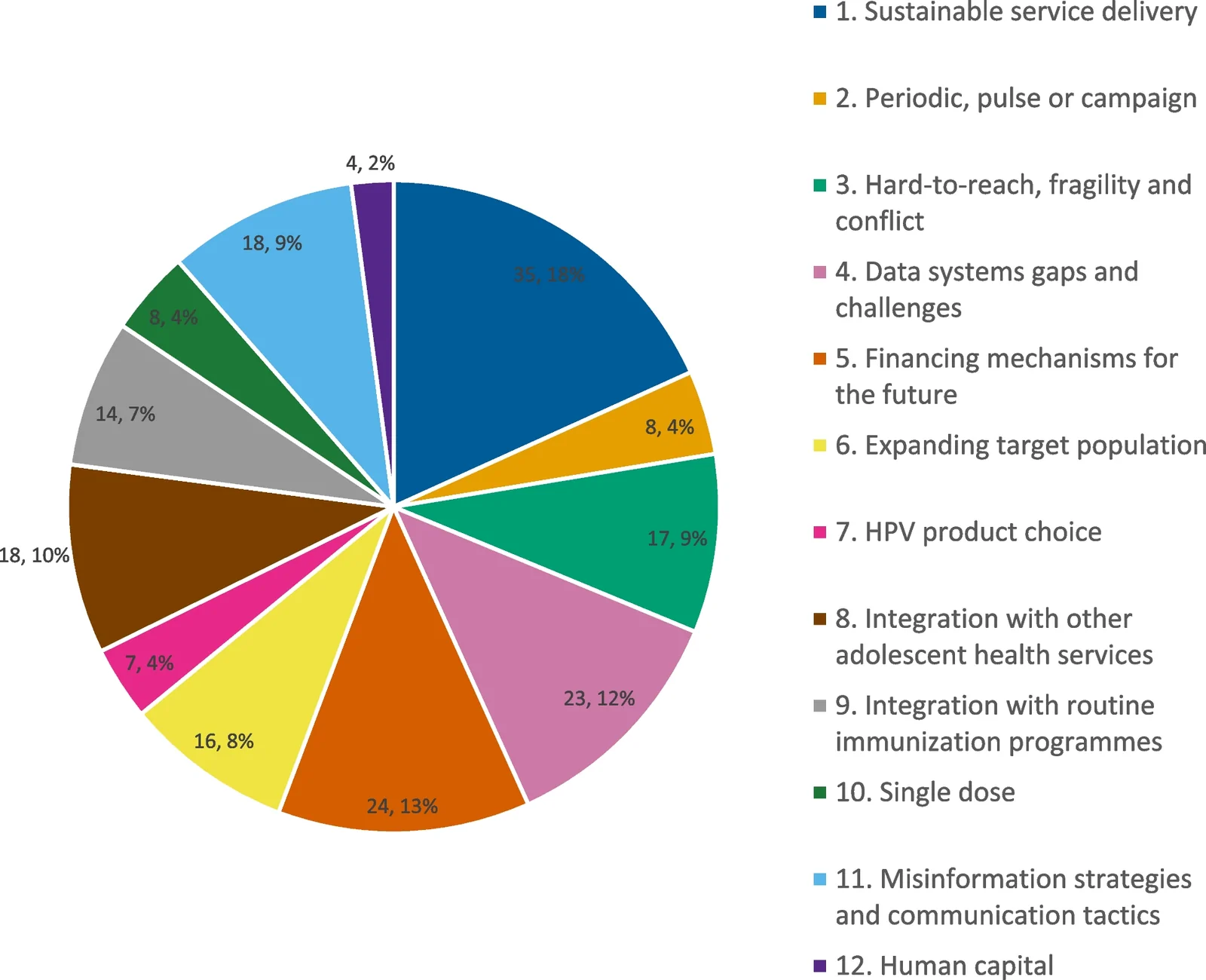
Distribution of participant voting preferences by theme (absolute number of votes, percentage)

At the close of the second day the Secretariat administered an electronic poll to capture participant perspectives on priority implementation challenges in real-time. Participants were presented with 12 potential themes (Fig. 2), drawn from initial needs assessment input from planning phases, and refined to respond to emerging topics of discussion surfaced during the first two days of the symposium. Each participant was asked to cast 3 votes to identify the themes most relevant to the HPV vaccination programs in their home country. The Secretariat analyzed these prioritization inputs, reducing the number of topics to focus the discussion and using them to inform participant allocation to working groups. This process also provided a snapshot of the program implementation issues felt to be most pressing across countries in 2025 (Fig. 3).

The poll captured 75 responses. We excluded 10 responses received from global partners and Advisors and removed two duplicates. The final analytical sample included 64 responses, 45 from anglophone countries and 19 from francophone countries, which together generated 192 individual votes across the 12 themes.

The theme of *sustainable service delivery* received the largest number of votes (35 out of 192 votes), while *financing (23/192 votes)* and *data systems* (24/192 votes) also ranked highly, followed by themes related to *hard-to-reach populations* and *service integration* (Fig. 3, Additional file 2 Table S2).

### Theme assignment

Once individual rankings, country of origin, and language had been taken into account, sixty participants were assigned to groups across 9 round table discussions for the first round; the same sixty were assigned to groups across 8 round table discussions for the second round (Table 2).

**Table 2:** Number of implementation working group round table participants by theme and discussion round

Theme	Round 1, English	Round 1, French	Round 2, English	Round 2, French
Sustainable service delivery	5 + 5 (2 groups)	10	7	–
Integration with routine immunization programs	6	–	–	–
Integration with other health services	7	–	7	–
Misinformation strategies and communication tactics	6	–	–	–
Data systems gaps and challenges	6	–	7	–
Hard-to-reach, fragility, and conflict	–	–	6	9
Financing mechanisms for the future	6	9	7	–
Expanding target populations	–	–	7	10
***Total, by Round***	***41***	***19***	***41***	***19***

We took care to keep the groups to a manageable size, although French-speaking groups were by necessity slightly larger to accommodate the limited number of available and qualified francophone moderators. Assignments were announced in plenary at the opening of the third day, and Secretariat members directed participants to their selected tables, where Advisors and other global partners provided facilitation and note-taking support to the discussions.

### Theme discussions

During both rounds, facilitators led each thematic working group through round-robin-style three-part discussions of: 1) the challenges and programmatic implications of the theme being discussed; 2) country-level interventions, successes, and good practices; 3) and resources, technical assistance, and other partnerships that might be needed for implementation of good practices and approaches to address the theme of the discussion (Additional file 2 Table S3). A rapid report-out plenary session at the end of the second round allowed participants a brief reflection period to consider additional perspectives and to share highlights from their own discussions with other peers around the room.

## Country priorities for action

The symposium concluded with a working group session that re-convened participants into their country contingents, to develop targeted action plans outlining the key steps needed to strengthen the HPV vaccination program in their home countries over the next year (Table 3). Each team developed these plans during a dedicated session on Day 3, using a structured process for reflection and collective application of peer learning discussions, particularly those during the preceding thematic working groups. Each of the 22 country-based groups reviewed the challenges they had collectively identified in their home country program, worked together to prioritise topics, and defined concrete actions, responsible stakeholders, and feasible timelines for implementation.

**Table 3:** Distribution of priority themes across country action plans

Theme		Countries
**Data systems improvement (see also **Table 4**)**		Cameroon, Kenya, Malawi, Mozambique, Nigeria, Tanzania, Uganda, Zambia
**Capacity building**		Côte D'Ivoire, The Gambia, Mali, Sierra Leone, Uganda
**Communication and community engagement**		Burkina Faso, Burundi, Cameroon, Côte D'Ivoire, The Gambia, Guinée, Kenya, Malawi, Namibia, Rwanda, Senegal
**Periodic, pulse or campaign**		Côte D'Ivoire, South Africa
**Expanding target populations**		Cameroon, Côte D'Ivoire, South Africa
**Sustainable Financing (see also **Table 5**)**		Burundi, Cameroon, Democratic Republic of the Congo, Guinée, Kenya, Malawi, Rwanda, Uganda, Zambia, Zimbabwe
**Strategies for hard-to-reach, fragility, and conflict**		Burkina Faso, Malawi, Mali, Mozambique, Namibia, Rwanda, Tanzania
**Integration with other adolescent health services**		Burkina Faso, Mali, Nigeria, Senegal, Sierra Leone, Uganda, Zimbabwe
**Sustainable Service Delivery**	Multisectoral coordination	Burkina Faso, The Gambia, Mali, Nigeria, Senegal, Sierra Leone, South Africa, Tanzania, Zambia
	Stakeholder engagement	Burundi, Democratic Republic of the Congo, Namibia, Nigeria, Uganda
	Planning and microplanning	Democratic Republic of the Congo, Guinée, Zambia
**Single dose transition**		Kenya^1^

^1^ At the time of the symposium activities (early October 2025), Kenya was in the process of transitioning from a two-dose to a single-dose HPV vaccination schedule. The transition was formally implemented later in November 2025

A representative from each country team briefly presented the highlights of their action plan to the symposium in a plenary session, and each country team submitted a written action plan worksheet. The Secretariat subsequently analyzed action plans to surface common and notable thematic priorities across countries. While specific actions varied by context, the most common actionable themes included: strengthening data systems; improving misinformation strategies, communication tactics and community engagement; ensuring sustainable service delivery, and securing sustainable financing mechanisms for the future.

Several countries also outlined actions related to integration of HPV vaccination with other adolescent health services; developing and implementing strategies for reaching hard-to-reach populations, and expanding target populations for vaccine eligibility and outreach. One country, Guinée, included actions to support HPV vaccine introduction to their national immunization program. Action item themes presented below reflect areas of convergence across countries rather than details of individual country plans.

Many of the countries' actions related to data, though countries referred to very different types of data challenges and system needs. To reflect these differences, we grouped the data-related actions into five areas: (A) data quality and use, which focuses on improving the accuracy, reporting, and interpretation of HPV vaccine data; (B) target population estimation, which strengthens identification and enumeration of eligible girls; (C) digitalisation, covering shifts from paper to electronic tools; (D) data integration, linking HPV vaccine data with other systems such as HIV or school records; and (E) forecasting, which improves tools for estimating vaccine needs and supporting micro-planning (Table 4).

**Table 4:** Data-related action areas and country distribution

Country	A. Data Quality / Use	B. Target Estimation	C. Digitalisation	D. Data Integration	E. Forecasting
Cameroon	–	Use school enrolment register for target estimation; integrate into school calendar	–	–	Update forecasting tool to include HIV +, catch-up groups, and boys
Kenya	Strengthen monitoring and evaluation through digitalisation; improve interoperability	Clearly define denominators (10-year-old girls)	Digitisation of immunization data	–	–
Malawi	Strengthen HPV data management (registers, cards); monitor HPV delivery	–	–	–	–
Mozambique	–	–	Digital integration implied (EPI + CC systems)	Integrate EPI and cervical cancer elimination systems into one	–
Nigeria	Improve data quality; strengthen cancer registry	–	–	Integrate HPV vaccination data with HIV and other databases; improve disaggregation	–
Tanzania	–	–	Roll out electronic immunization management system	Integrate HPV vaccination indicators, including for girls living with HIV	–
Uganda	Improve data quality and use; leverage data to target hard-to-reach areas	Improve target estimation for hard-to-reach populations	–	–	–
Zambia	–	–	–	Integrate HPV vaccination data with ART platforms for girls living with HIV	–

Actions related to financing mechanisms for the future were also very commonly included in country plans, though country groups referred to varied aspects of financial sustainability and system readiness. To capture this variation, we grouped the financing-related actions into five sub-domains: (A) domestic resource mobilisation, (B) political and financial advocacy, (C) budget planning and sustained funding mechanisms, (D) cost optimisation, and (E) partner engagement and external resource alignment. We have mapped each relevant country’s proposed actions to these categories, reflecting the diverse approaches countries intend to use to secure and protect financing for HPV vaccination (Table 5).

**Table 5:** Financing-related action areas and country distribution

Country	A. Domestic Resource Mobilisation	B. Political / Financial Advocacy	C. Budget Planning & Sustained Funding	D. Cost Optimisation	E. Partner Engagement / External Support
Burundi	Mobilising domestic resources for health	–	–	–	Mobilizing funds from other partners
Cameroon	–	–	Securing a budget line for procurement and operations	Switch from Gardasil 4 to less expensive quadrivalent	–
Democratic Republic of the Congo	Mobilisation of additional financial resources	–	–	–	–
Guinée	Mobilisation of local resources	Introduction championed by the Prime Minister (“vaccination champion”)	–	–	–
Kenya	–	Advocacy for immunization budget (national and subnational); stakeholder engagement; data-driven advocacy	–	–	–
Malawi	–	–	Integrated financing for HPV and routine vaccines	–	–
Rwanda	–	–	Plan sustainable financing and secure vaccine supply	–	High-level dialogues and partner coordination
Uganda	–	High-level advocacy for increased domestic funding for immunization and school health programs	–	–	Engage civil society organizations, PATH/CHAI, partners
Zambia	–	Advocacy for increased domestic funding; local government advocacy	–	–	Civil society, community leaders, Ministry of Finance
Zimbabwe	–	Advocate for increased domestic funding; political leaders and religious institutions	Strengthen public–private partnerships (as financing model)	–	Private sector engagement

## Synthesis of symposium proceedings

Our synthesis of discussions across the meeting, including the content-rich outputs gathered from the thematic working group peer learning discussions, yielded a number of cross-cutting observations that reflect shared implementation realities rather than isolated thematic concerns.

First, discussions related to decreasing HPV disease risk for people living with HIV surfaced in the majority of discussions. This challenge is felt and was noted across sustainable service delivery efforts, data systems, financing mechanisms, current global and local policy, and as part of conversations about expanding the populations of focus for HPV vaccination.

Second, Francophone countries in particular are interested in expanding the focus population for HPV vaccination. In peer learning discussions focused on expanding target populations, francophone country participants in particular described expanding vaccination to young and adult women living with HIV as the first step in an expansion effort. Discussions took an ethical view, expecting national leadership as well as donors to do the “right thing” and provide funding mechanisms to support such expansion. Discussions signalled that participants are eager for guidance from sectors beyond the vaccine world on how to optimise vaccination programs to serve this – and other – priority populations.

Third, peer learning discussions across most themes reflected that data systems are fragmented, with paper records at point of service and slow-to-adapt digital systems at the registry and aggregate level. This creates multiple levels of double work, and with increasing pressure to integrate service delivery programs, system interoperability is not keeping pace. Programmatic risks of fragmented data systems include those to confidentiality and privacy of persons living with HIV needing additional doses, and misrepresentation and under-resourcing of already-underserved communities who may be poorly enumerated at baseline.

Fourth, the topic of integration was discussed in multiple ways across a number of different thematic working groups, and the Secretariat notes the role that CHIC is poised to play in supporting the dissemination, and translation to policy and programs, of the growing body of evidence on how and why integrated approaches might work in a given context, particularly outputs that are anticipated from the Gavi HPV Learning Portfolio starting in 2026 [10].

Fifth, financing was likewise a recurring topic throughout the meeting, featuring prominently in the agenda, the thematic working group discussions, and the country action plans. Participants highlighted concerns about the long-term sustainability of HPV vaccination programs in the context of constrained domestic budgets, evolving donor support, and increasing expectations for program integration. Discussions frequently emphasised that financing considerations cut across service delivery, data systems, population targeting, and integration efforts, shaping both what is programmatically feasible and how quickly countries can act.

Sixth, although optimizing communication strategies to mitigate mis- and dis-information was not ranked among the highest priority themes during voting, their recurrent emergence across plenary discussions, breakout groups, and country action plans suggests a disconnect between formal prioritisation and perceived operational risk. The depth and engagement observed during the misinformation-focused breakout group indicate that participants view misinformation, particularly in the context of social media and emerging AI-generated content, as an evolving challenge that spans multiple programmatic areas rather than a standalone thematic issue. This pattern implies that communication challenges may be under-recognised in prioritisation exercises, despite being widely experienced as a practical constraint on implementation and sustainability of HPV vaccination programs.

Finally, across countries, HPV program sustainability emerged as closely tied to broader socioeconomic and structural conditions shaping health system performance. Limited fiscal space and competing health priorities constrain long-term financing, while gaps in digital infrastructure and uneven education-sector capacity affect the reach and continuity of adolescent vaccination delivery. These constraints are often compounded by urban–rural inequities, fragile governance environments, and differing levels of domestic financing capacity, reinforcing the need for sustainability approaches that are tailored to country context. Taken together, the findings suggest that durable HPV vaccination programs depend not only on technical design but on strengthened multisectoral governance and closer integration between health, education, and community delivery platforms to equitably reach adolescents.

### Next steps

In order to continue building upon and advancing peer-to-peer learning exchanges throughout the year, next steps for engagement across the CHIC community of practice include plans for communication forums between symposia – typically webinars scheduled throughout the year to focus on programmatic challenges with emerging learnings or evolving consensus on best practice. The Secretariat implements a refreshed communication strategy starting in the months after each symposium that includes dissemination of posters, slide decks, and other materials presented by participants at the in-person symposium; dissemination of responsive technical advocacy products prepared by the Secretariat to help close gaps in evidence synthesis identified by our community; and continued engagement with key partners to help advance the process of translating new evidence into practice, such as planned interactions and collaborations with the HPV Vaccine Acceleration Program Partners Initiative (HAPPI), Gavi HPV Learning Agenda partners, Future Evidence, Unicef, WHO, and others.

## Conclusion

This fifth, regional symposium advanced the CHIC contribution to fostering and growing the HPV vaccine community of practice, providing an effective platform for peer-to-peer learning and exchange of timely knowledge on emerging evidence and good practices. CHIC forums enable countries in various stages of HPV vaccine introduction to share experiences, identify common barriers, and plan proactive, informed solutions to sustain advances in vaccine access. Evidence from across the region continues to demonstrate both remarkable progress and persistent structural challenges, underscoring the importance of sustained political commitment, financial transition planning, digital system strengthening, and equity-focused strategies in pursuit of the WHO global strategy to eliminate cervical cancer by 2030.

## Supplementary Information

Additional file 1.

Additional file 2.

## Data Availability

The presentations, posters, and country snapshots shared during the symposium are publicly available on the CHIC website (stophpv.org). Any additional information related to this report is available from the corresponding author upon reasonable request.

## Abbreviations

AI: Artificial Intelligence
ART: Antiretroviral Therapy
CC: Cervical cancer
CHIC: Coalition to Strengthen the HPV Immunization Community
EPI: Expanded Program on Immunization, now called Essential Program on Immunization
ESA: Eastern and Southern Africa
HAPPI: HPV Vaccine Acceleration Program Partners Initiative
HCW: Healthcare Worker
HIV: Human Immunodeficiency Virus
HMIS: Health Management Information System
HPV: Human Papillomavirus
HPV1: First-dose HPV vaccine coverage
HPVc: Completed-series HPV vaccine coverage
IPVS: International Papillomavirus Society
MAC: Multi-Age Cohort
NA: Not Applicable
OOS: Out of School
PATH: Program for Appropriate Technology in Health
SMS: Short Message Service
UNICEF: United Nations Children's Fund
WHO: World Health Organization
